# Supplementary material for: Content-rich biological network constructed by mining PubMed abstracts
Source: BMC Bioinformatics. 2004 Oct 8;5:147. doi: 10.1186/1471-2105-5-147 (PMC528731; doi:10.1186/1471-2105-5-147)
Supplement: Additional File 2 — The original results of the above study (non-essential files are deleted to keep the file size under the limit set by BMC bioinformatics). [file 1471-2105-5-147-S2.bz2 › chilibotAdditionalFile2/dip10/299ID7486683E1290/html/RASA1_PDGFRB.html]

 


 **RASA1** and **PDGFRB** 
  
Found 28 abstracts in PubMed, retrieved 10.  
 

 What does Google say? 
 PDF only 
| .edu only 

---

**Interactive relationship** (e.g. stimulation, inhibition, etc)

**Neutral relationship**- RasGAP  [ **RASA1** ] , that responds to activation of PDGFR  [ **PDGFRB** ]  beta but not PDGFR  [ **PDGFRB** ]  alpha, was not phosphorylated on tyrosine in mutant cells upon PDGF treatment.  Ref: 9696037 Oncogene, 1998
- On ligand induced tyrosine phosphorylation, PDGFR  [ **PDGFRB** ]  associated with phospholipase C PLC gamma 1, Ras GTPase activating protein  [ **RASA1** ]  RasGAP  [ **RASA1** ] , and phosphatidylinositol 3 kinase PI3K but not with the Syp growth factor receptor bound protein 2 Son of Sevenless complex.  Ref: 9612285 Am J Physiol, 1998
- We have found that the F2 mutation has only a modest effect on ligand dependent activation of a chimeric PDGFR  [ **PDGFRB** ]  composed of the extracellular and transmembrane domains of the alphaPDGFR and the cytoplasmic domain of the betaPDGFR by three measures 1 the ability to phosphorylate endogenous and exogenous protein substrates in vitro, 2 phosphorylation of tyrosine 857, and 3 binding of the effector proteins PLCgamma, RasGAP  [ **RASA1** ] , and SHP 2.  Ref: 11281646 Exp Cell Res, 2001

**Non-interactive relationship** (e.g. studied together, co-existance, homology, etc.)

- Furthermore, we demonstrate that p62 dok can exert its negative effect on the PDGFR  [ **PDGFRB** ]  MAPK pathway independently of its ability to associate with RasGAP  [ **RASA1** ]  and Nck.  Ref: 11489946 J Exp Med, 2001
- PDGF stimulated the association of PDGFR  [ **PDGFRB** ]  beta with p85, ras GTPase activating protein  [ **RASA1** ]  and PLC gamma.  Ref: 10697503 Anticancer Res< MedlineTA>Anticancer Res, 2001
- Association of PDGFR  [ **PDGFRB** ]  with PLC gamma 1 and RasGAP  [ **RASA1** ]  followed by their tyrosine phosphorylation failed to activate PLC gamma 1, protein kinase C PKC, and Ras.  Ref: 9612285 Am J Physiol, 1998
